# Simultaneous resection of a neuroendocrine tumor in an incidental Meckel’s diverticulum with transabdominal preperitoneal hernial repair: a case report

**DOI:** 10.1186/s40792-024-01821-0

**Published:** 2024-01-17

**Authors:** Shoko Kato, Takuya Saito, Shintaro Kurahashi, Yasuyuki Fukami, Shunichiro Komatsu, Kenitiro Kaneko, Tsuyoshi Sano

**Affiliations:** Department of Surgery, Aichi Medical University, 1-1 Yazakokarimata, Nagakute, Aichi 480-1195 Japan

**Keywords:** Meckel’s diverticulum, Neuroendocrine tumor, Transabdominal preperitoneal repair

## Abstract

**Background:**

As laparoscopic surgery becomes more prevalent worldwide, Meckel’s diverticula are increasingly being discovered incidentally during surgery. There is no consensus on whether to follow up or resect such diverticula, which are usually asymptomatic. In cases of transabdominal preperitoneal inguinal hernia repair, resection of such a diverticulum might add the risk of mesh infection. Thus, it is unclear whether simultaneous intestinal resection is advisable.

**Case presentation:**

A 64-year-old man diagnosed with a left indirect inguinal hernia underwent laparoscopic inguinal hernia repair, during which a 2-cm Meckel’s diverticulum located contralateral to the mesentery of the ileum approximately 30 cm from Bauhin’s valve was detected incidentally. Because of the potential risk of future complications such as hemorrhage, diverticulitis, or tumor development, wedge resection of the ileum was performed extracorporeally through an extended umbilical port site after completion of the hernia repair. Pathological examination revealed a neuroendocrine tumor (G1) in Meckel’s diverticulum, which was successfully resected without any mesh infection or postoperative complications.

**Discussion:**

Our patient’s clinical course raises two important issues. First, a Meckel’s diverticulum detected incidentally during laparoscopic surgery should be resected promptly because malignant tumors within such diverticula have frequently been reported. Second, simultaneous resection with hernia repair using mesh seems to be as safe as other clean-contaminated surgery.

**Conclusions:**

Management of incidental Meckel’s diverticula should be selected by appropriate assessment for the risk of malignancy and complications.

## Background

Inguinal hernias are common worldwide and surgical procedures for them have developed significantly over the past few decades. Transabdominal preperitoneal (TAPP) hernia repair has become the preferred procedure in many medical centers because it is minimally invasive and often achieves positive outcomes. With the increasing adoption of laparoscopic approaches, there has been an increasing trend toward incidental findings during surgery, a Meckel’s diverticulum being a relatively common example. However, a clear consensus or guideline for the optimal management of an incidentally detected Meckel’s diverticulum during laparoscopic surgery has yet to be established [1–5].

We here report a rare case of Meckel’s diverticulectomy performed after the TAPP procedure. Histopathological examination of the operative specimen resulted in diagnosis of a neuroendocrine tumor (NET).

## Case presentation

A 64-year-old man was referred to our hospital for a swelling in the left inguinal region. He reported no other symptoms and his medical history was unremarkable. Computed tomography showed a left inguinal hernia with no other findings. Preoperative laboratory tests were normal. We decided to perform TAPP hernia repair in accordance with our institution’s policies.

**Fig. 1:**
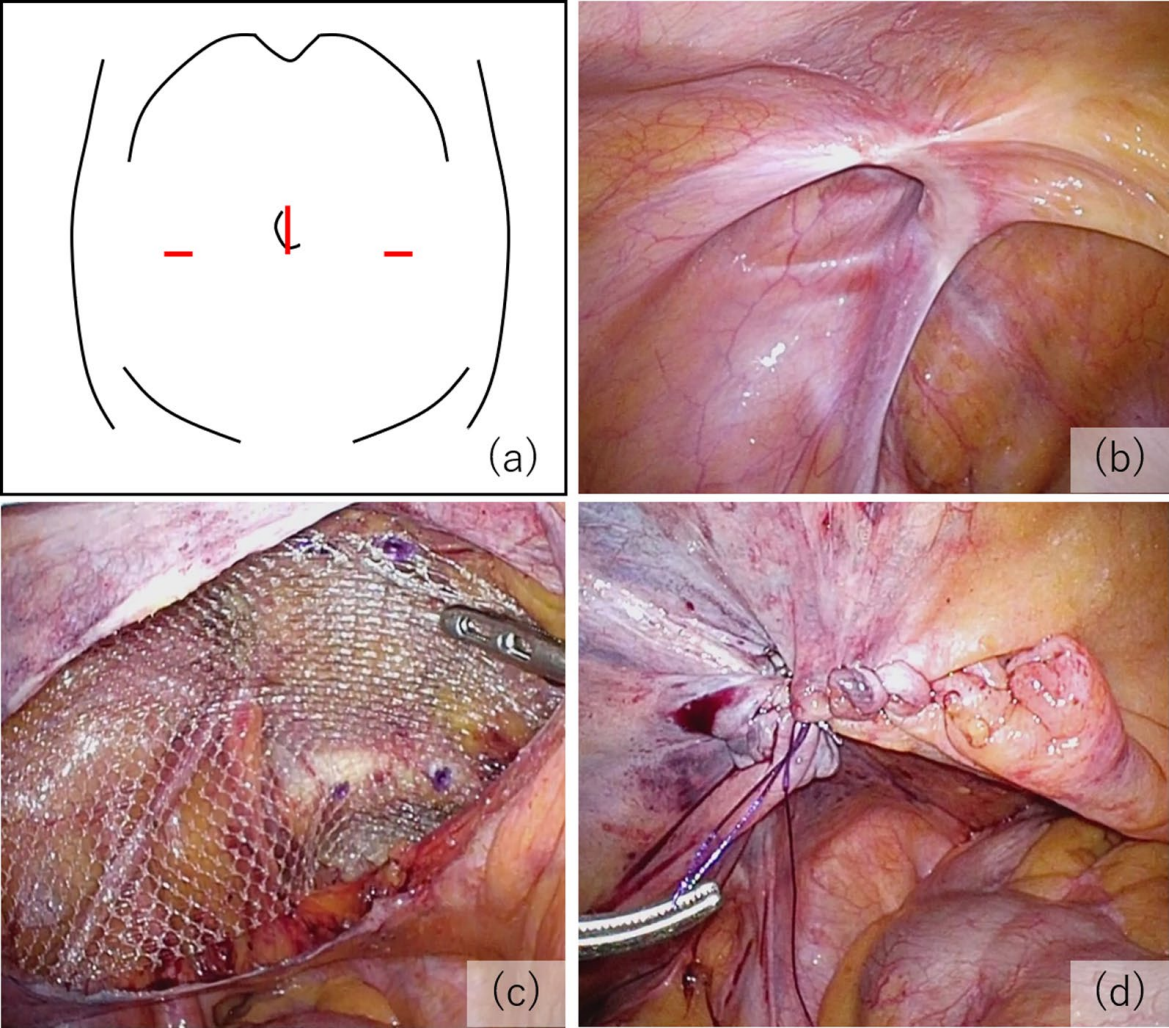
Transabdominal preperitoneal inguinal hernia repair. **a** Port arrangement. The umbilical port was 12 mm and the others 5 mm. **b** The hernial orifice, which corresponds to EHS-L2. **c** Mesh fixation. **d** Closure of the peritoneal flap

**Fig. 2:**
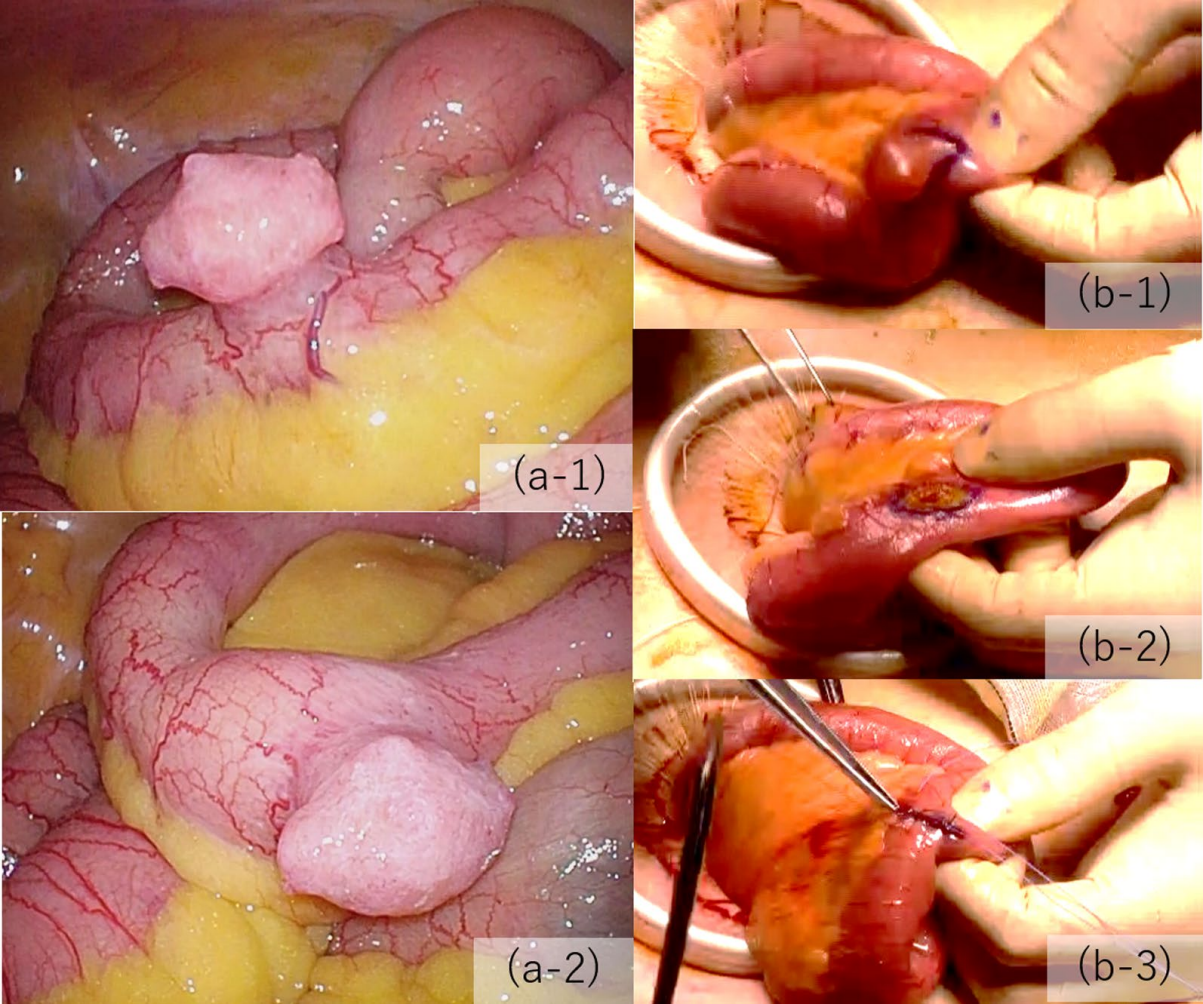
Meckel’s diverticulectomy. **a** Photograph of an ileal diverticulum. The surface has some bulges and resembles a tumor. **b** Wedge resection of the ileum

**Fig. 3:**
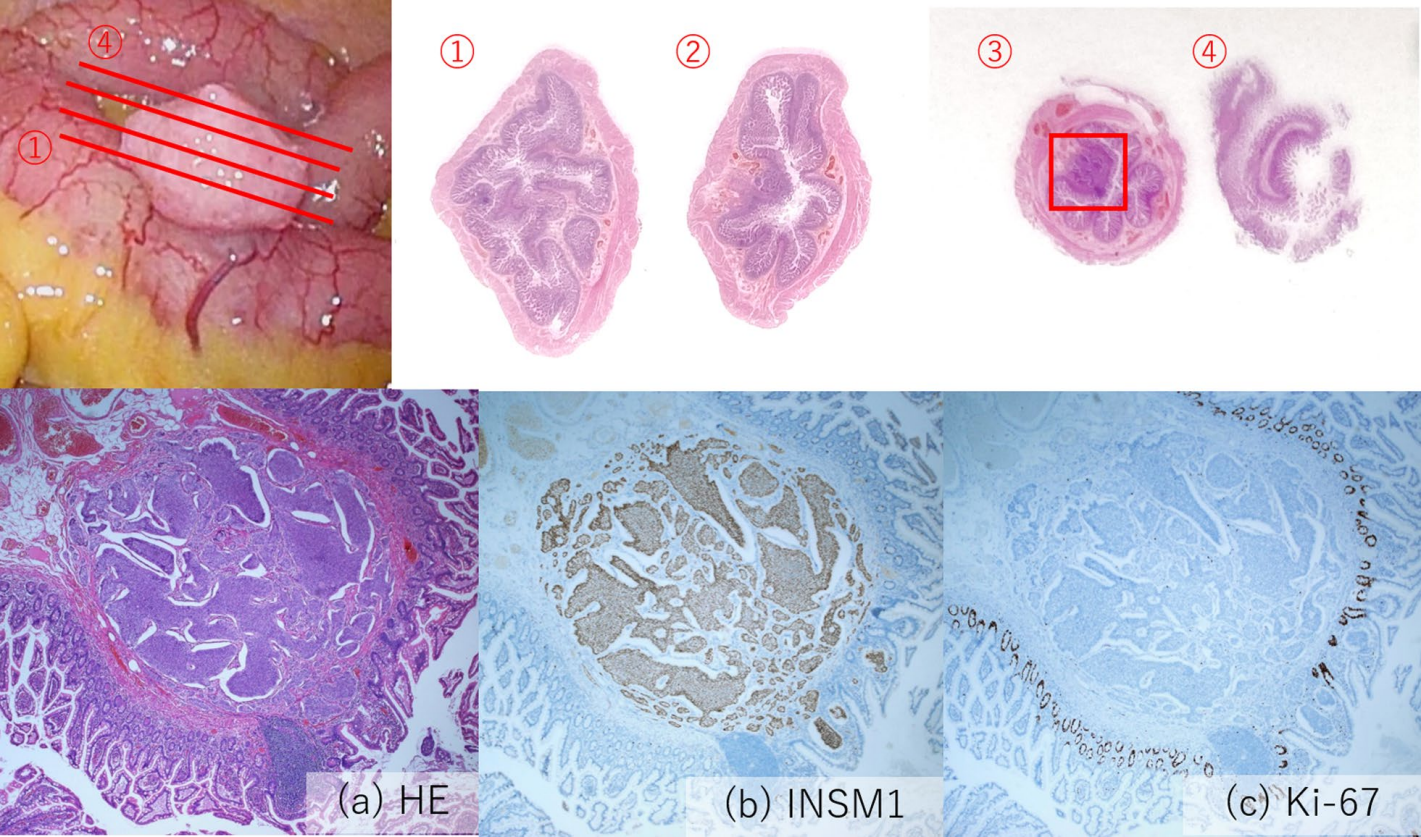
Pathological findings. The tumor (red square) exists in the submucosal layer of Meckel’s diverticulum. **a** Photomicrograph showing a solid tumor. **b** Photomicrograph showing the tumor in the submucosal layer is positive for INSM1. **c** Photomicrograph showing Ki-67-positive cells account for < 1% of all cells

We established three ports, one at the umbilicus and the other two in the lateral abdominal region on each side (Fig. 1a). During laparoscopic inspection, we found a 2-cm diverticulum located contralateral to the mesentery of the ileum approximately 30 cm from Bauhin’s valve (Fig. 2a). It was not inflamed and had a narrow base and some surface irregularities resembling a tumor; there was no evidence of peritoneal dissemination. Because of the potential risk of future complications, such as hemorrhage, diverticulitis, or tumor development, we decided to resect it after fixation of mesh and closure of the peritoneal flap. The hernial orifice was 2 cm in diameter and diagnosed as L2 according to the European hernia society groin hernia classification [6] (Fig. 1b). After making sufficient space in the preperitoneal cavity, we fixed 15 × 10 cm of polypropylene mesh (Bard^®^ Soft Mesh; Becton–Dickinson, Franklin Lakes, NJ, USA) with poly (lactic-co-glycolic acid) tacker (AbsorbaTack^®^; Medtronic, Minneapolis, MN, USA) (Fig. 1c). Thereafter, the umbilical port site was extended and wedge resection of the ileum performed extracorporeally (Fig. 2b). The diverticulum was wholly soft and removed along minimally invasive line. The ileum was sutured by Gambee technique. The pathological diagnosis was a NET (G1) in a Meckel’s diverticulum (Fig. 3). The tumor was 2 mm in diameter, invaded the submucosal layer, and positive for INSM1 (insulinoma-associated protein 1). Ki-67 was positive in < 1% of cells. No ectopic mucosa was detected. The postoperative course was uneventful with no mesh infection or postoperative complications. No further therapy was recommended because the tumor was quite small and the Ki67 labeling index low. The patient has had no recurrence in three years of follow-up.

## Discussion

The clinical course of this patient raises two important issues. One, that a Meckel’s diverticulum detected incidentally during laparoscopic surgery should be resected promptly; and two, that simultaneous surgery of Meckel’s diverticulectomy and TAPP hernia repair is safe.

First, a Meckel’s diverticulum detected incidentally during laparoscopic surgery should be resected without delay because there have been frequent reports of malignant tumors in Meckel’s diverticula. Indeed, our patient had a NET within his Meckel’s diverticulum. Eighty-four percent of malignancies in Meckel’s diverticulum have been reported to be NETs [7]. NETs are also known to have a high rate of lymph node metastasis [8, 9]. In particular, NETs with submucosal invasion in the jejunum or ileum (less than 5 mm) have a 17.2% rate of metastasis to lymph nodes, which is significantly higher than NETs in the rectum or stomach [9]. In Japan, only 9.6% of gastrointestinal NETs are in the midgut [10]. In contrast, in other countries 30–60% of gastrointestinal NETs are in the midgut [10, 11]. Among NETs in the midgut, only six cases of NETs in a Meckel’s diverticulum have been reported in Japan [12]. However, all six of these NETs were symptomatic (pain, fever, or ileus) and diagnosed postoperatively, suggesting that the true prevalence may be higher. Even asymptomatic Meckel’s diverticula without malignancy can result in various long-term complications such as diverticulitis, gastrointestinal bleeding, and perforation [2, 13]. The decision to resect a Meckel’s diverticulum should be made with consideration of both the frequency of long-term postoperative complications and risk of malignancy [3, 14–17]. However, we consider resection essential to definitely minimizing the risk of malignancy.

Second, simultaneous TAPP hernia repair and Meckel’s diverticulectomy can be performed safely provided appropriate infection-control procedures are in place. The main concern with simultaneous surgery is the risk of hernia mesh infection. Some papers published before 2010 reported that bowel resection could result in postoperative infection of hernial mesh [18]. However, studies published after 2015 suggest that simultaneous hernial operation with another regional operation, such as colorectal surgery [19], cholecystectomy [20, 21], or prostatectomy [22], does not increase mesh infection. Actually, 14 cases of simultaneous hernial repair and another contaminated surgery (six resection of gastrointestinal lesion, four laparoscopic cholecystectomy, two hepatectomy, one distal pancreatectomy, one lymph node dissection in the pelvis) in our institution from 2016 to 2023 did not cause mesh infection.

To date, there have been few reports of simultaneous TAPP hernia repair and Meckel’s diverticulectomy. In one case similar to ours [23], the authors reported that laparoscopic resection of incidental Meckel’s diverticulum using an endoscopic linear stapler is safe. In a neonate with an incidental Meckel’s diverticulum discovered during a hernia repair, edge resection of the Meckel’s diverticulum was performed four days after laparoscopic hernia repair [24]. Provided the risk of mesh infection is adequately managed, simultaneous surgery is advisable because it minimizes the physical and economic burden on the patient. The sequence during surgery is important in minimizing mesh contamination. We suggest that hernia repair should precede bowel resection and the mesh must be completely covered by a peritoneal flap (Fig. 1d) before bowel manipulation to prevent contamination mediated by surgical instruments [20], thus allowing simultaneous surgery to be performed efficiently and safely.

## Conclusions

We here report a patient in whom a NET was discovered after resection of an incidental Meckel’s diverticulum following TAPP hernia repair. In conclusion, simultaneous surgery can be considered safe. Further case reports are needed in the future.

## Data Availability

During preparation of this report, no data were analyzed, reused, or generated.

## Abbreviations

INSM: Insulinoma-associated protein
NET: Neuroendocrine tumor
TAPP: Transabdominal preperitoneal
